# Commentary: Increased CDC6 Expression Associates With Poor Prognosis in Patients With Clear Cell Renal Cell Carcinoma

**DOI:** 10.3389/fonc.2021.730782

**Published:** 2021-10-05

**Authors:** Mustafa Zafer Temiz

**Affiliations:** Department of Urology, Bagcilar Training and Research Hospital, Istanbul, Turkey

**Keywords:** CDC6, prognosis, clear cell renal cell carcinoma, survival, pathway

## Introduction

I read with great interest the recent article by Yicong et al. (1) on the association of tissue cell division cycle 6 (CDC6) expression with the prognosis for clear cell renal cell carcinoma (ccRCC). I would like to congratulate the authors for their valuable work and interesting study and wish to highlight some issues.

In the study, the authors investigated primarily the relationship between CDC6 expression and standard pathological features and prognosis with tissue immunohistochemistry in 118 ccRCC patients. They showed that CDC6 expression was higher in ccRCC tissue samples compared to that in normal kidney tissues. The study also indicated that the overall survival (OS) of the patients in the low CDC6 group was longer compared to that of the patients in the high CDC6 group. CDC6 expression was closely related to some prognostic parameters, including age, tumor size, T stage, and Fuhrman grade, in tissue obtained from 118 patients. Similarly, the expression levels of CDC6 were associated with grade, race, and stage in ccRCC patients from The Cancer Genome Atlas (TCGA) database. The authors concluded that ccRCC patients with a high expression of CDC6 are more likely to develop advanced disease than do ccRCC patients with a low CDC6 expression. Additionally, the study conducted univariate and multivariate Cox regression analyses by using TCGA dataset to investigate the prognostic role of CDC6 expression in terms of OS. In both analyses, CDC6 expression was found to be an independent risk factor for the prognosis of ccRCC patients.

## Commentary and Discussion

Although the authors have proposed a potential predictive marker for tumor stage and the prognosis of ccRCC patients, it seems that some of the other clinicopathological parameters have been ignored. Today, undoubdetly, Fuhrman nuclear grading and the TNM systems are the most important prognostic parameters for RCC. However, they have several limitations and are still not perfect (2). To improve prediction of the prognosis of ccRCC patients, several clinicopathological parameters other than nuclear grading and tumor stage systems—such as age, gender, performance status of the patients, tumor location, lymphovascular invasion (LVI), sarcomatoid and rhabdoid features, tumor necrosis, tumor growth pattern (expansive or infiltrative), warm ischemia time, multifocality and/or bilateral occurrence of carcinoma, and caval or renal thrombosis—have been used in clinical urology practice (2–5). It is my belief that the best inferences on the prognostic role of a potential marker might be derived with more comprehensive univariable and multivariable analyses. Parameters other than age, race, and stage, such as gender, performance status, LVI, and sarcomatoid and rhabdoid features, could have been considered in Cox analyses. Similarly, especially in tissue obtained from 118 ccRCC patients rather than from TCGA dataset, investigation of the differentially expressed CDC6 levels for the patients with LVI, sarcomatoid and rhabdoid features, and local recurrence and distant metastasis might have enriched the study. After the gene set enrichment analysis (GSEA) study and online TIMER and protein–protein interaction (PPI) network analyses, it was also found that a high expression of CDC6 was related to multiple signaling pathways, immune checkpoint molecules, tumor microenvironment, and immune infiltration in the study. It was previously reported that various inflammation-based prognostic parameters, e.g., platelet-to-lymphocyte ratio (PLR) and neutrophil-to-lymphocyte ratio (NLR), and other markers of elevated systemic inflammation might be useful for predicting survival of patients with several malignant neoplasms and RCC (6–8). In this regard, the relationship between CDC6 tissue expression and some of those blood markers provided by retrospective patient data analysis could have been investigated and could have strengthened the study.

Finally, the use of cancer-specific survival and local recurrence and distant metastases besides OS as other prognostic parameters during the analyses would have provided more clear inferences. Moreover, it would contribute to the research on cRCC therapies in the era of needing additional targeted therapies or predictors of current therapeutic algorithms for advanced cRCC (9). For instance, the programmed death-ligand 1 (PD-L1) protein, which exhibits prognostic value for various malignancies, had been investigated for its predictive value during the management of advanced RCC. The results revealed significantly greater overall and complete response rates in PD-L1-positive *versus* PD-L1-negative patients [odds ratio (OR) = 1.84, 95% CI = 1.48–2.28; OR = 3.11, 95% CI = 2.04–4.75, respectively] (9, 10). In this regard, in the near future, CDC6 expression will probably serve as a novel predictor in this subject. Who knows.

Respectfully.

## Author Contributions

The author confirms being the sole contributor of this work and has approved it for publication.

## Conflict of Interest

The author declares that the research was conducted in the absence of any commercial or financial relationships that could be construed as a potential conflict of interest.

## Publisher’s Note

All claims expressed in this article are solely those of the authors and do not necessarily represent those of their affiliated organizations, or those of the publisher, the editors and the reviewers. Any product that may be evaluated in this article, or claim that may be made by its manufacturer, is not guaranteed or endorsed by the publisher.
